# Impact of adherence to procalcitonin antibiotic prescribing guideline recommendations for low procalcitonin levels on antibiotic use

**DOI:** 10.1186/s12879-022-07923-0

**Published:** 2023-01-19

**Authors:** Brian E. Malley, Jonathan G. Yabes, Elizabeth Gimbel, Chung-Chou H. Chang, Donald M. Yealy, Michael J. Fine, Derek C. Angus, David T. Huang, for the ProACT Investigators, Yohei Doi, Tammy L. Eaton, Michael J. Fine, Elizabeth A. Gimbel, Octavia M. Peck Palmer, Francis Pike, Ashley M. Ryman, Lisa A. Weissfeld, Kourtney A. Wofford, Tianyuan Xu, Jonathan G. Yabes, Michael W. Donnino, Imoigele P. Aisiku, Peter C. Hou, Raghu R. Seethala, Robert L. Sherwin, John M. Holst, Michelle A. Fischer, Colleen M. Rafferty, William D. Dachman, Frank LoVecchio, Michael R. Filbin, Michael K. Mansour, Jonathan M. Fine, Jean M. Hammel, Matthew J. Exline, Lauren T. Southerland, Thomas E. Terndrup, Michael C. Kurz, David L. McCullum, Henry E. Wang, Alpesh N. Amin, Shahram Lotfipour, Feras H. Khan, R. Gentry Wilkerson, Heather A. Prunty, Brian Suffoletto, Aaron M. Brown, Franziska F. Jovin

**Affiliations:** 1 The CRISMA (Clinical Research, Investigation, and Systems Modeling of Acute Illness) Center, University of Pittsburgh, Pittsburgh, PA USA; 2 Department of Critical Care Medicine, University of Pittsburgh, Pittsburgh, PA USA; 3 Division of General Internal Medicine, University of Pittsburgh, Pittsburgh, PA USA; 4 Department of Emergency Medicine, University of Pittsburgh, Pittsburgh, PA USA; 5 Center for Health Equity Research and Promotion, VA Pittsburgh Healthcare System, Pittsburgh, PA USA; 6 The MACRO (Multidisciplinary Acute Care Research Organization) Center, University of Pittsburgh, Pittsburgh, PA USA; 7 University of Pittsburgh, 606B Scaife Hall, 3550 Terrace Street, Pittsburgh, PA 15261 USA

**Keywords:** Procalcitonin, Antibiotic, Guideline adherence

## Abstract

**Background:**

The Procalcitonin Antibiotic Consensus Trial (ProACT) found provision of a procalcitonin antibiotic prescribing guideline to hospital-based clinicians did not reduce antibiotic use. Possible reasons include clinician reluctance to follow the guideline, with an observed 64.8% adherence rate. In this study we sought to determine the threshold adherence rate for reduction in antibiotic use, and to explore opportunities to increase adherence.

**Methods:**

This study is a retrospective analysis of ProACT data. ProACT randomized 1656 patients presenting to 14 U.S. hospitals with suspected lower respiratory tract infection to usual care or provision of procalcitonin assay results and an antibiotic prescribing guideline to the treating clinicians. We simulated varying adherence to guideline recommendations for low procalcitonin levels and determined which threshold adherence rate could have resulted in rejection of the null hypothesis of no difference between groups at alpha = 0.05. We also performed sensitivity analyses within specific clinical settings and grouped patients initially prescribed antibiotics despite low procalcitonin into low, medium, and high risk of illness severity or bacterial infection.

**Results:**

Our primary outcome was number of antibiotic-days by day 30 using an intention-to-treat approach and a null hypothesis of no difference in antibiotic use. We determined that an 84% adherence rate in the hospital setting (emergency department and inpatient) for low procalcitonin could have allowed rejection of the null hypothesis (3.7 vs 4.3 antibiotic-days, p = 0.048). The threshold adherence rate was 76% for continued guideline adherence after discharge. Even 100% adherence in the emergency department alone failed to reduce antibiotic-days. Of the 218 patients prescribed antibiotics in the emergency department despite low procalcitonin, 153 (70.2%) were categorized as low or medium risk.

**Conclusions:**

High adherence in the hospital setting to a procalcitonin antibiotic prescribing guideline is necessary to reduce antibiotic use in suspected lower respiratory tract infection. Continued guideline adherence after discharge and withholding of antibiotics in low and medium risk patients with low procalcitonin may offer impactful potential opportunities for antibiotic reduction.

*Trial registration* Procalcitonin Antibiotic Consensus Trial (ProACT), ClinicalTrials.gov Identifier: NCT02130986. First posted May 6, 2014.

## Background

Procalcitonin is a circulating peptide typically elevated in bacterial but not viral infection. Several European randomized trials found that provision of a procalcitonin antibiotic prescribing guideline to hospital-based clinicians safely reduced antibiotic use in suspected lower respiratory tract infection [1–4]. These trials reported high clinician guideline adherence, with the largest reporting a 79.3% adherence rate [3]. We conducted the Procalcitonin Antibiotic Consensus Trial (ProACT) to determine generalizability in a contemporaneous U.S. cohort and found no overall reduction in antibiotic use. Of the possible reasons for this finding, the lower clinician adherence rate of 64.8% compared to the earlier trials may explain much of the varying impact [5]. In addition, decisions to initially withhold antibiotics based on the procalcitonin guideline may have been subsequently overruled in the outpatient setting [6].

We sought to determine what guideline adherence rate to antibiotic withholding recommendations at low procalcitonin levels would have resulted in less antibiotic use in the ProACT trial and in which settings. We also explored how clinically challenging it would be for clinicians to achieve guideline adherence based on predictors of illness severity and bacterial infection.

## Methods

ProACT was a patient-level, 1:1 randomized trial in 14 hospitals in the United States. We enrolled 1656 adult patients presenting with an initial diagnosis of acute lower respiratory tract infection. Treating clinicians estimated the likelihood of bacterial etiology before any experimental actions. Patients randomly received usual care (no other interventions or guides) or an intervention where the treating clinicians received procalcitonin results and an antibiotic use guideline with graded recommendations based on four tiers of procalcitonin levels. The guideline used the same cutoff values as in previous trials and as approved by the U.S. Food and Drug Administration (antibiotics strongly discouraged for procalcitonin levels < 0.1 mcg/L, discouraged for levels 0.1 to 0.25 mcg/L, recommended for levels > 0.25 to 0.5 mcg/L, and strongly recommended for levels > 0.5 mcg/L). On discharge, we provided patients with a letter for their primary care provider that included their last procalcitonin assay result, a trial synopsis, and the procalcitonin guideline. The primary outcome was total antibiotic exposure to day 30 and data were obtained through chart review and by telephone calls at days 15 and 30 made by coordinating center staff who were unaware of the treatment-group assignments. Using baseline characteristics and published criteria, we categorized the initial diagnosis of lower respiratory tract infection into final diagnoses of acute exacerbation of chronic obstructive pulmonary disease (COPD), asthma exacerbation, acute bronchitis, community-acquired pneumonia, and other.

### Statistical analyses

To evaluate the impact of adherence on the primary outcome of antibiotic-days by day 30, we varied adherence between 65% and 100% and determined which threshold adherence rate for low procalcitonin levels (≤ 0.25 mcg/L) in the intervention arm could have allowed rejection of the null hypothesis (no difference between groups) at a significance level of 0.05. The usual care control arm (n = 830) was used for comparison and was not altered in simulations. In the procalcitonin intervention arm we identified all patients who had guideline nonadherent antibiotic prescriptions. To examine 100% adherence, we set antibiotic use to zero at each timepoint when procalcitonin levels were under guideline cutoffs in all intervention arm patients (n = 826). To examine adherence rates < 100%, we simulated an intervention arm population of the same size (n = 826) with the given adherence rate. To account for variability in sampling, we present the mean results of repeating this sampling process 1000 times.

To create each simulated intervention arm population we first included all patients who had only guideline-adherent antibiotic prescriptions. We then used random selection without replacement in R version 3.6 to sample the requisite number of patients with nonadherent prescriptions needed to simulate the given adherence rate. For each of these sampled patients we set their antibiotic use to zero for the timepoints where their procalcitonin levels were below the guideline cutoff. For the patients with nonadherent prescriptions who were not selected and for the patients with only guideline-adherent prescriptions we used their observed antibiotic-days. As with the original report of the ProACT trial, we compared the mean number of antibiotic-days between usual care and intervention groups using two-sample t-tests, following an intention-to-treat approach with multiple imputation to account for patients lost to follow-up after discharge.

Our primary analysis focused on the protocol period in the hospital including both emergency department (ED) and inpatient, where clinicians received procalcitonin results. In sensitivity analyses, we also determined the threshold adherence rate for rejection of the null hypothesis for adherence only in the emergency department, or in the hospital (ED and inpatient) for only for the lowest procalcitonin levels (< 0.1 mcg/L). These analyses addressed whether hospital clinician adherence to procalcitonin guidance only in the emergency department, or only for the lowest procalcitonin levels, could be sufficient to reduce antibiotic use.

We also determined the threshold rate for continued guideline adherence after discharge. For this analysis, we selected patients whose last procalcitonin level prior to discharge was low and who did not receive antibiotics on the day this procalcitonin level was reported. This analysis addressed whether avoidance of overruling in the outpatient setting of initial decisions to withhold antibiotics could be sufficient to reduce antibiotic use.

To explore the second question of how challenging it would be to achieve adherence, we examined the characteristics of patients presenting with a low procalcitonin who were initially prescribed antibiotics on presentation in the emergency department. We then grouped these patients into low, medium, and high risk of illness severity or bacterial infection based on published risk criteria and the treating clinician’s estimate of the likelihood of bacterial etiology [7–9]. Our intent was to characterize patient scenarios where the decision to withhold antibiotics would be difficult (high risk), reasonable (low risk), or intermediate (medium risk).

We categorized as high risk all patients with a new infiltrate on chest imaging or > 1 risk criterion of illness severity or bacterial infection. We defined risk criteria based on the systemic inflammatory response syndrome (SIRS), quick Sepsis Related Organ Failure Assessment (qSOFA), the Center for Medicare and Medicaid Services SEP-1 criteria, baseline demographics (age, Charlson comorbidity score), clinician estimate of likelihood of bacterial etiology, oxygen saturation, and hospitalization status [7–9]. We limited low risk patients to only those without a new chest infiltrate and with no risk criteria. Thus, we deemed only younger patients with no chest imaging infiltrate, discharged home from the emergency department, with normal vital signs, mental status and oxygen saturation, lower comorbidity burden, and lower clinician estimate of likelihood of bacterial etiology as low risk. We considered medium risk all other patients—i.e., patients without a new chest infiltrate and with at most 1 risk criterion (Appendix Table 3).

## Results

**Fig. 1:**
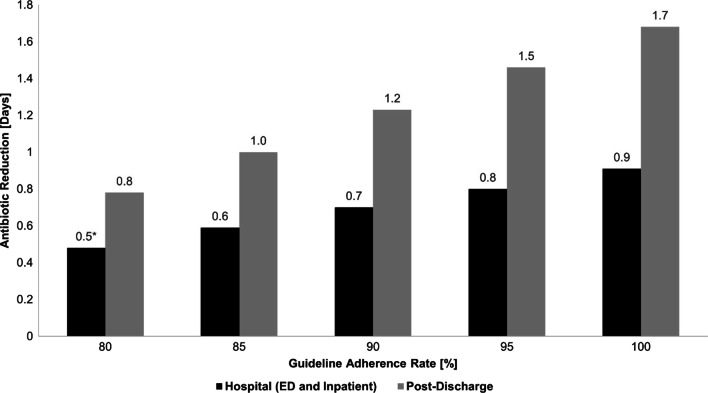
Reduction in antibiotic-days to day 30 by procalcitonin guideline adherence rate by different epochs of care. This figure shows the reduction in antibiotic days to day 30 for a given simulated procalcitonin guideline adherence rate for the hospital (combined emergency department and inpatient) and post-discharge epochs of care. *Indicates that the reduction in antibiotic days was not significant. All other reductions were significant at an alpha of 0.05

The threshold hospital (ED and inpatient) adherence rate for rejection of the null hypothesis was 84% for low procalcitonin levels in intervention arm patients (3.7 days, vs 4.3 days, − 0.6 day difference, p = 0.048) (Table 1). Complete guideline adherence in the hospital (100%) would have resulted in 0.9 fewer antibiotic-days (Table 1, Fig. 1). There was no scenario in which adherence only in the emergency department would have resulted in less antibiotic use. Even 100% adherence resulted in a non-significant reduction (Table 2). For adherence to the lowest procalcitonin levels in intervention arm patients, the threshold rate was 91% (Table 2).

**Table 1:** Number of antibiotic-days by day 30 for varying in-hospital (emergency department and inpatient) adherence to low procalcitonin level (≤ 0.25 mcg/L) antibiotic withholding guidance

Adherence	Usual care Mean (SD) n = 830	Procalcitonin Mean (SD) n = 826	Difference (95% CI)	p-value
100%^a^	4.3 (5.6)	3.4 (5.4)	− 0.9 (− 1.8, − 1.5)	0.001
95%	4.3 (5.6)	3.5 (5.5)	− 0.8 (− 0.8, − 0.8)	0.005
90%	4.3 (5.6)	3.6 (5.5)	− 0.7 (− 0.7, − 0.7)	0.015
85%	4.3 (5.6)	3.7 (5.6)	− 0.6 (− 0.6, − 0.5)	0.040
80%	4.3 (5.6)	3.8 (5.6)	− 0.5 (− 0.5, − 0.4)	0.094
75%	4.3 (5.6)	3.9 (5.7)	− 0.4 (− 0.4, − 0.3)	0.191
70%	4.3 (5.6)	4.0 (5.7)	− 0.3 (− 0.3, − 0.2)	0.352
65%	4.3 (5.6)	4.1 (5.8)	− 0.2 (− 0.2, − 0.1)	0.579

^a^Except for 100% adherence, values presented are averaged across 1000 resampled datasets

*SD* standard deviation, *CI* confidence interval

**Table 2:** Number of antibiotic-days by day 30 for varying adherence to antibiotic withholding guidance for the lowest procalcitonin level (≤ 0.1 mcg/L) in-hospital (inpatient and emergency department), low procalcitonin level (≤ 0.25 mcg/L) in the emergency department only, and low procalcitonin level (≤ 0.25 mcg/L) post-discharge

Adherence	Procalcitonin ≤ 0.1 mcg/L ED and inpatient			Procalcitonin ≤ 0.25 mcg/L ED only			Procalcitonin ≤ 0.25 mcg/L Post-discharge		
	Mean Days	Difference from usual care (95% CI)	p-value	Mean Days	Difference from usual care (95% CI)	p-value	Mean Days	Difference from usual care (95% CI)	p-value
100%^a^	3.6	− 0.7 (− 1.6, − 1.3)	0.011	4.0	− 0.3 (− 1.2, − 0.9)	0.273	2.6	− 1.7 (− 2.5, − 2.2)	< 0.001
95%	3.6	− 0.6 (− 0.7, − 0.6)	0.026	4.0	− 0.3 (− 0.3, − 0.3)	0.328	2.8	− 1.5 (− 1.5, − 1.4)	< 0.001
90%	3.7	− 0.6 (− 0.6, − 0.5)	0.052	4.0	− 0.3 (− 0.3, − 0.2)	0.388	3.1	− 1.2 (− 1.3, − 1.1)	< 0.001
85%	3.8	− 0.5 (− 0.5, − 0.4)	0.100	4.1	− 0.2 (− 0.2, − 0.2)	0.454	3.3	− 1 (− 1.1, − 0.9)	< 0.001
80%	3.9	− 0.4 (− 0.4, − 0.4)	0.173	4.1	− 0.2 (− 0.2, − 0.2)	0.526	3.5	− 0.8 (− 0.9, − 0.7)	0.006
75%	4.0	− 0.3 (− 0.4, − 0.3)	0.282	4.1	− 0.2 (− 0.2, − 0.1)	0.605	3.7	− 0.6 (− 0.7, − 0.5)	0.052
70%	4.1	− 0.2 (− 0.3, − 0.2)	0.430	4.2	− 0.1 (− 0.1, − 0.1)	0.688	N/A	N/A	N/A
65%	4.1	− 0.2 (− 0.2, − 0.1)	0.616	4.2	− 0.1 (− 0.1, − 0.1)	0.774	N/A	N/A	N/A

^a^Except for 100% adherence, values presented are averaged across 1000 resampled datasets. All procalcitonin arm patients (n = 826) were used for each analysis

*SD* standard deviation, *CI* confidence interval

Of patients with low procalcitonin levels in whom antibiotics were initially withheld, 36.7% (172 of 469) subsequently received antibiotics after discharge. The threshold adherence rate to reduce antibiotic use was 76% for continued guideline adherence after discharge (Table 2). Complete guideline adherence after discharge (100%) would have resulted in 1.7 fewer antibiotic-days (Table 2, Fig. 1). Threshold adherence rates for continued guideline adherence after discharge varied across lower respiratory tract infection type (community-acquired pneumonia—76%; acute exacerbation of chronic obstructive pulmonary disease—75%; acute bronchitis—86%; asthma exacerbation—87%, Appendix Table 4).

Of the 218 patients presenting with a low procalcitonin who were prescribed antibiotics in the emergency department, 17 (7.8%) were categorized as low, 136 (62.4%) as medium, and 65 (29.8%) as high risk of illness severity or bacterial infection.

## Discussion

The impact of a novel diagnostic test depends on the characteristics of the healthcare environment in which it is implemented. Efforts to study and implement novel tests should examine the entire epoch of care from initial encounter through the outpatient period to fully characterize the impact of testing on care. Our main finding was that a high adherence rate to procalcitonin guidance in the hospital setting (ED and inpatient) would have been necessary to observe a reduction in antibiotic use in the ProACT trial. Our results also indicated that continuing guideline adherence post-discharge could have a greater impact on antibiotic reduction, with more than one in three patients with low procalcitonin and no initial antibiotic prescription later receiving antibiotics after discharge.

Withholding antibiotics can be a source of anxiety to both clinicians and patients. Both groups may be more willing to forego antibiotics when procalcitonin is low and clinical risk factors are low to moderate versus when procalcitonin is low but clinical risk is high. Our simulations demonstrated that continuing guideline adherence after discharge would have resulted in the greatest reduction in antibiotic use, and that a large proportion of patients prescribed antibiotics despite low procalcitonin levels had low to moderate risk of severe disease. We found that of patients initially prescribed antibiotics despite low procalcitonin, less than one in three were deemed to be at high risk of severe disease or bacterial etiology. These patients represent a potentially amenable target for improving adherence to a procalcitonin antibiotic prescribing guideline either in limiting initial prescriptions or for earlier cessation of antibiotics.

Complete guideline adherence is generally unachievable in medicine and may not even be desirable as it would eliminate clinical judgment. These issues raise the question of what level of adherence is achievable and reasonable to target. In the ProACT trial procalcitonin guidelines were implemented using quality improvement principles and adherence varied from 39.5% to 83.3% between centers [6]. Our simulations found threshold hospital adherence rates higher than that observed at any ProACT site or in prior trials of procalcitonin prescribing guidelines [3]. It is unlikely we would observe a higher rate of guideline adherence in general clinical practice than seen in multiple controlled trial environments. However, outpatient procalcitonin testing was not included in the ProACT trial as procalcitonin is only approved for hospital (ED and inpatient) use in the United States. Our simulations indicate that continued post-discharge adherence would have reduced antibiotic use even at lower adherence rates than in prior trials. However, to avoid reversals in the outpatient setting of previous decisions to withhold antibiotics in patients with low procalcitonin, the initial test results and guideline would need to be readily available after discharge, and outpatient clinicians would need to be able to retest to allay concerns of initial “false negative” procalcitonin cases and development of bacterial infection after discharge.

Our results are limited to the setting of the ProACT trial including patients with suspected lower respiratory tract infection and using serial testing. Results are also limited to patients and sites similar to those enrolled in ProACT; relatively young, non-critically ill patients with low comorbidity burden, presenting to predominantly urban tertiary care academic centers with low baseline antibiotic use. We assumed no harm would have resulted from lower antibiotic use due to increased adherence, as prior procalcitonin trials that resulted in lower antibiotic use found no harm [1–4]. Additionally, it is possible the modest reduction in antibiotic-days at the threshold adherence rates may not be clinically significant. We utilized broad criteria for patients at increased risk of severe illness or bacterial infection based on prior studies but these factors are neither exhaustive nor definitely positively predictive [7–9]. We took a conservative approach and did not exclude nonadherent antibiotics given prior to return of procalcitonin results so there is a possibility the hospital (ED and inpatient) threshold adherence rate is actually higher than our simulated result.

## Conclusions

Our simulations demonstrate that high adherence in the hospital (ED and inpatient) to a procalcitonin antibiotic prescribing guideline is necessary to reduce antibiotic use in suspected lower respiratory tract infection. Continued guideline adherence after discharge and withholding of antibiotics in low and medium risk patients with low procalcitonin may offer impactful potential opportunities for antibiotic reduction.

## Data Availability

The datasets generated during and/or analyzed during the current study are available from the corresponding author on reasonable request.

## Appendix

See Tables 3 and 4.

**Table 3:** Risk criteria of illness severity or bacterial infection

**High risk**	
New infiltrate on chest imaging, or ≥ 2 of any of the below risk criteria	
*SIRS/qSOFA/SEP-1*:	
Abnormal white blood cell count (> 12,000/µL or < 4,000/µL, or with greater than 10% bands)	
Abnormal temperature (< 36 or > 38 Celsius)	
Elevated heart rate (> 90)	
Elevated respiratory rate (≥ 22)	
Altered mental status	
Hypotension (systolic blood pressure < 90 or mean arterial pressure < 65 mmHg)	
*Baseline demographics*:	
Age ≥ 65 years	
Charlson comorbidity score > 2(8)	
*Other*:	
Clinician estimate of likelihood of bacterial etiology > 50%	
Oxygen saturation < 90%	
Hospitalized	
**Low risk**	
No new infiltrate on chest imaging, AND	
No risk criteria of illness severity or bacterial infection (as above)	
**Medium risk**	
All other patients	

*SIRS* systemic inflammatory response syndrome, *qSOFA* quick Sepsis Related Organ Failure Assessment, *SEP-1* Center for Medicare and Medicaid Services sepsis measure

**Table 4:** Adherence to post-discharge continuation of initial antibiotic withholding in patients with low procalcitonin levels (< 0.25 mcg/L) in the hospital, and impact on antibiotic-days by Day 30, by lower respiratory tract infection type

Adherence	# of antibiotic-days by Day 30; mean (SD)		Difference^a^	p-value^a^
	Procalcitonin^a^	Usual care		
a. Community-acquired pneumonia				
100%	4.6 (4.9)	7.2 (6.0)	− 2.5	< 0.001
95%	4.9 (5.2)	7.2 (6.0)	− 2.3	< 0.001
90%	5.1 (5.4)	7.2 (6.0)	− 2.1	0.002
85%	5.3 (5.6)	7.2 (6.0)	− 1.8	0.008
80%	5.5 (5.8)	7.2 (6.0)	− 1.6	0.021
75%	5.8 (6.0)	7.2 (6.0)	− 1.4	0.055
b. Chronic obstructive pulmonary disease				
100%	3.3 (4.3)	5.2 (5.3)	− 1.9	< 0.001
95%	3.5 (4.5)	5.2 (5.3)	− 1.8	< 0.001
90%	3.6 (4.7)	5.2 (5.3)	− 1.6	0.001
85%	3.8 (4.9)	5.2 (5.3)	− 1.4	0.004
80%	4.0 (5.1)	5.2 (5.3)	− 1.2	0.016
75%	4.2 (5.3)	5.2 (5.3)	− 1.0	0.045
c. Acute bronchitis				
100%	1.7 (4.0)	3.6 (5.5)	− 1.9	< 0.001
95%	2.0 (4.4)	3.6 (5.5)	− 1.6	0.002
90%	2.2 (4.7)	3.6 (5.5)	− 1.3	0.013
85%	2.5 (4.9)	3.6 (5.5)	− 1.1	0.053
d. Asthma				
100%	2.2 (3.9)	3.6 (4.9)	− 1.4	< 0.001
95%	2.4 (4.1)	3.6 (4.9)	− 1.1	0.003
90%	2.6 (4.3)	3.6 (4.9)	− 0.9	0.017
85%	2.9 (4.5)	3.6 (4.9)	− 0.7	0.071
80%	3.1 (4.7)	3.6 (4.9)	− 0.5	0.214
75%	3.3 (4.9)	3.6 (4.9)	− 0.3	0.489

^a^Except for 100% adherence, values presented are averaged across 1000 resampled datasets

*SD* standard deviation

## Abbreviations

ED: Emergency Department
ProACT: Procalcitonin Antibiotic Consensus Trial
qSOFA: Quick Sepsis Related Organ Failure Assessment
SIRS: Systemic inflammatory response syndrome
mcg/L: Micrograms per liter
